# Traditional Chinese medicine for cancer?

**DOI:** 10.1038/bjc.2012.282

**Published:** 2012-07-24

**Authors:** E Ernst

**Affiliations:** 1 Complementary Medicine, Peninsula Medical School, University of Exeter, Veysey Building, Salmon Pool Lane, Exeter EX2 4SG, UK

When faced with a diagnosis of cancer, many patients feel they must leave no stone unturned and try alternative treatments, such as traditional Chinese medicine (TCM) (Ernst and Cassileth, 1998). Yet oncologists often know little about such therapies. In this issue, Meng *et al* (2012) report a randomised clinical trial of a TCM preparation as an adjunctive therapy for advanced pancreatic adenocarcinoma. This study is remarkable in several respects.

The TCM preparation used was an intravenous extract from toad skin, which has a long tradition of use in China. Most oncologists might think that testing such a bizarre and exotic remedy must be a waste of time. Yet, as the authors of the trial (Meng *et al*, 2012) explain, the remedy is far less implausible than we might have assumed. In fact, it is backed up by *in-vitro* experiments, animal studies and preliminary clinical data. Thus, our knee-jerk reaction to dismiss such a trial out of hand would have been entirely inappropriate.

It has been shown repeatedly that studies of TCM tend to be of poor methodological quality and that Chinese investigators never report negative results (for example, Vickers *et al*, 1998). If true, this phenomenon would seriously distort the overall picture of TCM. The trial by Meng *et al* (2012) seems to indicate that things are changing. First, their study seems fairly rigorous, and second, their results fail to show that the TCM preparation is effective.

Meng *et al* (2012) rightly point out that their findings cannot be a final verdict on TCM. The chosen dose of their particular extract might have been too low; the treatment period might have been too short; other types of cancer might respond differently, and of course, the preparation of toad skin is by no means the only cancer therapy TCM has to offer. In fact, there are hundreds, if not thousands, of different TCM remedies traditionally used to treat cancer.

The task of sorting out the wheat from the chaff in TCM is therefore enormous. As Meng *et al* (2012) have shown, it would be a mistake to assume that a long history of use is a reliable indicator for efficacy. To advance our knowledge, we need reliable data rather than anecdotes, hunches or reports about a long history of usage. It would be surprising, if amongst the many TCM preparations we would not find some valuable compounds. But to make progress, we need to conduct research, and we need to do it systematically and rigorously – everything else would be irresponsible and not in our patients’ best interest.
